# Examining Letter Detector Tolerance through Offset Letter Halves: Evidence from Lexical Decision

**DOI:** 10.5334/joc.322

**Published:** 2023-09-27

**Authors:** Manuel Perea, Inka Romero-Ortells, Melanie Labusch, María Fernández-López, Ana Marcet

**Affiliations:** 1 Department of Methodology and ERI-Lectura, Universitat de València, Valencia, Spain; 2 Centro de Investigación Nebrija en Cognición (CINC), Universidad Nebrija, Madrid, Spain; 3 Department of Basic Psychology, Methodology, and ERI-Lectura, Universitat de València, Valencia, Spain; 4 Department of Psychology and Sociology, School of Social and Human Sciences, Universidad de Zaragoza, Teruel, Spain; 5 Grupo de Investigación en Enseñanza de Lenguas (GIEL), Department of Language and Literature Teaching, Universitat de València, Valencia, Spain

**Keywords:** lexical access, word recognition, lexical decision, letter distortion

## Abstract

Neurobiological models of reading assume that the specialized detectors at the letter level (e.g., the arrays of detectors for the letter ‘n’) possess a certain degree of tolerance (e.g., Local Combination Detectors model, Dehaene et al. 2005). In this study, we designed two lexical decision experiments that examined the limits of tolerance of letter detectors by introducing a novel manipulation involving shifting letter halves (e.g., 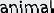 in Experiment 1; 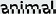 in Experiment 2) relative to intact items. This manipulation alters the transition between upper and lower parts of the letters, adding junctions that do not exist in the intact letter forms. We included high- and low-frequency words in the stimulus list to investigate whether letter distortion affects processing beyond the letter level, reasoning that interactive effects would signal top-down lexical feedback. In Experiment 1, which employed a subtle letter shift, we observed a minimal cost of letter distortion that did not interact with word frequency. Experiment 2, employing a larger letter shift, revealed an overall greater reading cost that affected differentially high- and low-frequency words. Overall, these findings offer insights into the limits of resilience in letter detectors to distortion during word recognition and introduce a novel manipulation of letter distortion.

One of the most remarkable feats of human cognition is our capacity to read efficiently under suboptimal scenarios (see Grainger, 2018, 2022; Grainger & Dufau, 2012, for reviews). We can effortlessly read words even if the visual appearance of their constituent letters is less than perfect with only a small cost, as in the case of words written with poor handwriting (e.g., 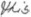; see Barnhart & Goldinger, 2010; Qiao et al., 2010; Vergara-Martínez et al., 2021), words with rotated letters (e.g., 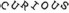; see Blythe et al., 2019; Fernández-López et al., 2021; Kim & Straková, 2012), words with digit-like (“leet”) letters (e.g., M4T3R14L; see Molinaro et al., 2010; Perea et al., 2008), or even Captchas (e.g., 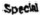, see Hannagan et al., 2012).

This small reading cost can be explained by neurobiological models of visual word recognition which, in the spirit of models of objects and face recognition (Riesenhuber & Poggio, 1999; Rolls, 2000), propose a hierarchical structure of layers of detectors with increasing complexity and broader receptive fields (e.g., Local Combination Detectors [LCD] model, Dehaene et al., 2005; see also Grainger et al., 2008). These detectors range from various layers of neurons that respond to letter contours, portions of letters, different letter forms, abstract letter representations, portions of words, and complete word units. One fundamental assumption of these models is that, while being selective, the detectors possess a certain level of tolerance to noise (see Riesenhuber & Poggio, 1999). For example, at the level of letter detectors, the neurons specialized in the letter “A” could also fire when the stimulus is the letter-like digit “4” in the word M4TR1X (Dehaene & Cohen, 2007; see also Marcet & Perea, 2018, for evidence of activation of muti-letter combinations, as in *harnburger*). Only when the visual word form is heavily distorted (e.g., 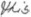) would increase attentional deployment help normalize the visual percept (Qiao et al., 2010; Vergara-Martínez et al., 2021; Vinckier et al., 2011).

Consistent with this view, previous lexical decision experiments have shown that easily-to-read handwritten words (e.g., 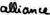) produce only slightly slower word identification times than printed words (e.g. alliance) regardless of whether the words’ frequency. This finding is consistent with the idea that letter detectors are barely affected by the small changes caused by the visual form of the handwritten words. Notably, difficult-to-read handwritten words (e.g., 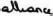) yield much longer word identification times than easy-to-read handwritten words and, furthermore, the effect of word frequency is amplified (see Barnhart & Goldinger, 2010; Perea et al., 2016). An explanation for this dissociative pattern is that when letter detectors cannot readily respond to the visual input of words, additional top-down feedback is required. In this context, feedback from the lexical level would benefit higher-frequency words more than lower-frequency words (Barnhart & Goldinger, 2010; see also Vergara-Martínez et al., 2021, for electrophysiological evidence). While intriguing, an intrinsic limitation of experiments with handwritten words is that the stimuli differ in various parameters (e.g., tilt, spacing, letter connectivity, variations in letter shape, among others).

The main aim of the present experiments was to examine the impact of a novel manipulation that tests the resilience of letter detectors to distortion in the visual word recognition system. This manipulation involves shifting half of each word’s letters (e.g., 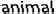). We compared the shifted condition to the intact condition, allowing us to obtain a measure of the reading cost. The shift was subtle (e.g., 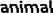 in Experiment 1) or moderate (e.g., 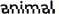 in Experiment 2). Shifting the letter halves disrupts the seamless transition between each letter’s upper and lower halves, resulting in fragmented lines and curves. Thus, this manipulation would hinder the mapping of the visual input onto the layers of letter units by breaking the continuity of curves and lines, creating junctions and vertices that do not exist in the prototypical letter forms stored in the word recognition system. Critically, we can combine this manipulation of letter distortion with a lexical factor such as word frequency (i.e., word recognition is faster for high- than low-frequency words). The logic is that if the effects of letter distortion and word frequency are additive, it would suggest that letter distortion primarily affects the letter level, equally impacting high- and low-frequency words. Alternatively, an interaction between these two factors would suggest the involvement of top-down processes from higher processing levels during word recognition.

To our knowledge, only one study has manipulated letter distortion by shifting the lower part of a word’s letters using a same-different perceptual task. Wilson and Taylor (2009; Experiment 3) employed a task where participants were required to determine whether two words presented for 40 ms sequentially, separated by a 520-ms blank interval, shared their upper halves. In the critical comparison, they contrasted pairs of words sharing the upper halves (e.g., 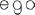 vs. 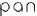) with pairs of words where the letters were shifted but still shared the upper halves (e.g., 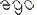 vs. 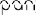).^1^ Wilson and Taylor (2009) found that participants had difficulty discriminating the intact pairs (58% of accuracy), but unsurprisingly, their performance improved for the pairs with the shifted letters (71% of accuracy). The authors concluded that this finding reinforced the role of whole letters as units during word recognition.

It is worth noting that previous studies have explored the effects of letter distortion (e.g., via missing letter features, letter parts, or by rotation of the word’s letters) on word recognition and reading. One line of research has examined this issue by examining the differential impact of omitting midsegments, vertices, or junctions during letter and word processing (e.g., see Fiset et al., 2009; Lanthier et al., 2009; Petit & Grainger, 2002; Rosa et al., 2016; Szwed et al., 2009). One shared finding in these studies is the importance of the letter’s junctions and vertices during word recognition, which is consistent with previous evidence in object recognition (e.g., see Biederman, 1987). Another line of research has focused on whether some parts of a word are more informative than others by omitting the upper or lower parts of words (Fiset et al., 2009; Perea, 2012). As anticipated by Huey (1908), the omission of the lower part of words is less damaging than the omission of the upper parts. And yet another line of research examined the impact of letter distortion by parametrically rotating the word’s constituent letters (Fernández-López et al., 2023; Kim & Straková, 2012). For instance, Fernández-López et al. (2023) found that when letter rotation is small (22.5º; e.g., 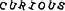), there is only a minimal reading cost relative to the intact words, which was additive to the word-frequency effect. Instead, for very high rotation angles (67.5º; e.g., 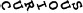), the reading cost was substantially larger, and low-frequency words were more affected by rotation angle than high-frequency words. This pattern suggests that, at these rotation angles, top-down lexical processes may normalize the identification of high-frequency words (Fernández-López et al., 2023).

In the present experiments, we directly manipulated the impact of letter distortion on word recognition by shifting the lower part of the word’s letters using a lexical decision task (i.e., the more standard word identification task). The items were presented in two basic formats: (1) intact (e.g., 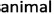), or (2) with the lower half of the word’s letters shifted (e.g.,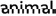 or 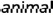) (see Figure 1, for depiction of the experimental conditions). We chose both directions (left and right) for the distorted letters so that participants could not anticipate the shift direction—in a parallel scenario, letter rotations produce similar costs regardless of whether they are clockwise or anti-clockwise (e.g., see Benyhe & Csibri, 2021, for recent evidence). Critically, the present manipulation of letter distortion allows for a parametrical examination of the amount of displacement and, therefore, the resistance of letter detectors to distortion. The shift was subtle in Experiment 1 (e.g., 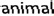) and moderate in Experiment 2 (e.g., 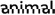, 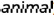)—this increase in letter distortion did not result in overlapping a given letter with its neighboring letters (see Figure 1).^2^ We also manipulated a lexical factor: word frequency. This allowed us to examine whether letter distortion mainly affected the letter level—this would be reflected in additive effects of the two factors—or whether it spilled over the lexical level.

**Figure 1:**
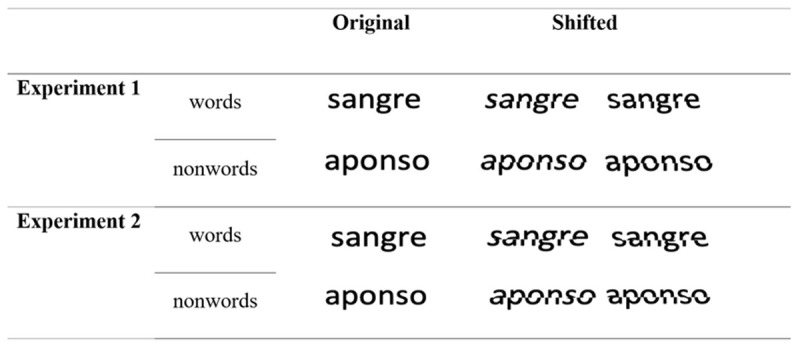
Example Stimuli of Experiments 1 and 2. Each item was presented either intact or shifted—the bottom half was displaced to the left or to the right. The Spanish word “sangre” is the English for blood.

It is important to note that letter rotation can also be manipulated parametrically (e.g., 0°, 22.5°, 45°; see Blythe et al., 2019; Fernández-López et al., 2021, 2023; Kim & Straková, 2012). Critically, unlike the current manipulation, where inner letter features are disrupted (e.g., by adding junctions and vertices that do not exist in the original items), letter rotation preserves these inherent features (e.g., 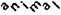). Indeed, the manipulation of letter rotation is more closely related to how the cognitive system encodes and decodes letter orientation and arrangement during lexical access. In contrast, the present manipulation specifically examines how disruptions in letter configuration within words impact lexical access.

The predictions for the experiments are as follows. If letter detectors in the visual-word recognition pathway tolerate, to some degree, variations in letter forms due to shifting the word’s lower parts, we should find a small reading cost exclusively at the letter level (LCD model, see Cohen & Dehaene, 2007). Thus, at least when the displacement is subtle (i.e., Experiment 1), the effect of letter distortion would be small and additive to the effect of word frequency. Alternatively, the alteration of the transition of the words’ upper and lower halves with the shifted items (e.g., junctions and vertices that do not occur in the intact letter forms) may add an extra burden to the letter detectors. This may be particularly so when the displacement is moderately large, as in Experiment 2. We must bear in mind that junctions are key features for models of letter and word recognition (see Szwed et al., 2009, for discussion). In this latter scenario, involvement from higher processing levels may be expected, leading to an interaction between the two factors.

## Experiment 1: Subtle shifts of the lower half

### Method

#### Participants

A total of 78 na tive individuals (mean age = 27.8 years, SD = 5.8 years) took part in the experiment (35 women). This sample size ensured at least 4212 observations in each format (original and shifted), which is above the guidelines of Brysbaert and Stevens (2018) for small-sized effects. All participants were native speakers of Spanish with normal (or corrected) vision and no reading or writing disorders. We recruited the participants via the online recruitment platform Prolific Academia (www.prolific.co), through which they received monetary compensation. The present study was approved by the Ethics Committee of Experimental Research at the University of Valencia, and all participants gave their informed consent before starting the experiment.

#### Materials

The stimulus set consisted of 162 Spanish words (81 categorized as high-frequency and 81 as low-frequency) and 162 pseudowords. All the stimuli were of six letters. Most items (160 words and 160 pseudowords) were used in Perea et al.’s (2018) Experiment 1. For the high-frequency words, the mean Zipf frequency, as determined by the subtitle EsPal database (Duchon et al., 2013), was 5.0 (range: 4.5–6.1). The mean Levenshtein distance of the 20 closest neighbors (OLD20) was 1.7 (range: 1.3–2.9), and the mean imageability rating on a 1–7 scale was 5.5 (range: 2.7–6.6). As for the low-frequency words, the mean Zipf frequency was 3.4 (range: 3.0–3.7), the mean OLD20 was 1.8 (range: 1.2–2.2), and the mean imageability was 5.3 (range: 3.0–6.6). High- and low-frequency words were matched in OLD20 and imageability (both *p*s > .15). The pseudowords were generated by Wuggy software (Keuleers & Brysbaert, 2010), following the orthotactic rules of Spanish.

The stimuli were presented in Calibri font, size 24-point, with black text on a white background. Each item was converted into an image. It could be presented either intact (e.g., 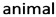) or with the lower half of the item’s letters 1 mm to the left or right (e.g., 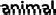 and 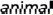). The shifted items were created using a Python program written for this purpose. As said earlier, we created two distorted versions for each item so that participants could not anticipate the direction of the shift. We created three lists to counterbalance the items across the three versions of the items. We randomly assigned participants to the three lists.

#### Procedure

The experiment was programmed with PsychoPy 3 (Peirce & MacAskill, 2021) and hosted online on the Pavlovia platform (www.pavlovia.org). In addition, the demographic data was collected via LimeSurvey (http://www.limesurvey.org). Participants were instructed to do the experiment in a quiet place without any distractions. The task was lexical decision: participants had to decide whether a string of letters was a word or not by pressing the m (“yes”) and z (“no”) buttons on their keyboard. We instructed participants to answer as quickly and accurately as possible. The program registered the response times and accuracy of the responses. A trial consisted of the presentation of a fixation cross for 50 ms and the presentation of the stimulus until a response was made (or until a deadline of 2000 ms). Before the start of the experiment, participants went through sixteen practice trials, receiving feedback for their responses. The experimental block did not contain feedback. There were 324 trials with breaks after every 80 trials, resulting in a median completion time of around 15 minutes.

### Results and Discussion

**Figure 2:**
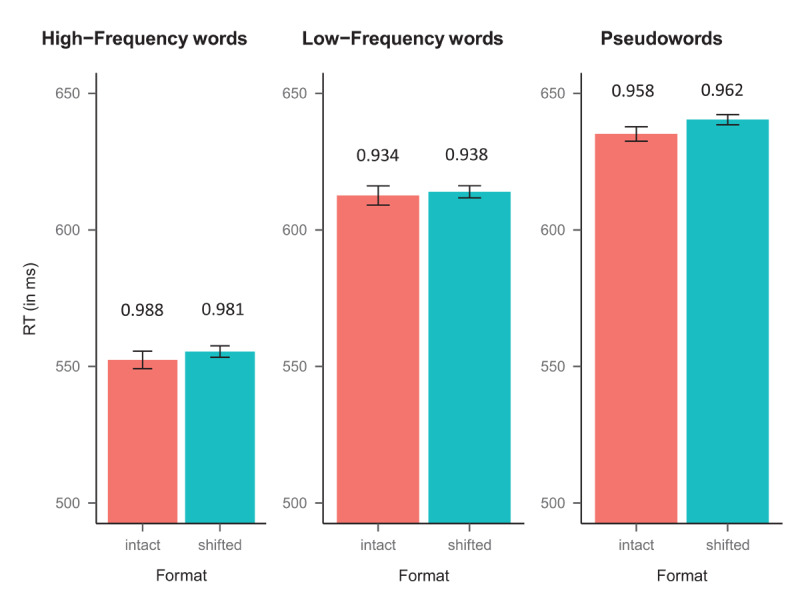
Mean response times (RTs, in ms) for the experimental conditions in Experiment 1. The error bars represent the standard errors, and the number above the bars correspond to the accuracy. *Note*: We observed larger reading costs, more akin to the model’s coefficients, for the harmonic means: (1) high-frequency words (529 vs. 534 ms for the intact and shifted words, respectively); (2) low-frequency words (585 vs. 590 ms for the intact and shifted words, respectively); and (3) pseudoword (604 vs. 610 ms for the intact and shifted pseudowords, respectively).

For the analyses of the response times, we excluded incorrect responses (4.0% for both words and nonwords) and extremely fast response times (<250 ms; 0.04% for words and 0.07% for nonwords). The left/right-shifted items were merged as one condition—for the interested reader, they behaved the same in the two experiments (see the OSF link at the end of this paper for these analyses). Figure 2 depicts the average response times (in ms) and accuracy in each experimental condition.

We employed brms (Bürkner, 2018) in R (R Core Team, 2022) to create Bayesian linear mixed-effects models on the word and nonword data for the inferential statistics. To model the integral positive asymmetry of response time distributions, we used the exgaussian distribution for the latency data, whereas to model the binary nature of accuracy data (1 = correct; 0 = incorrect), we used the Bernoulli distribution. For the word data, the fixed effects were word frequency (–0.5 for high-frequency words; 0.5 for low-frequency words) and letter distortion (–0.67 for intact [original] words; 0.33 for distorted [shifted] words)—the procedure was the same for the nonword data except that the only fixed factor was letter distortion. We chose the maximal random-effect structure in the design:


          RT [accuracy] = word_frequency * letter_distortion + (word_frequency *
 letter_distortion | subject) + (letter_distortion | item)
        

**Figure 3:**
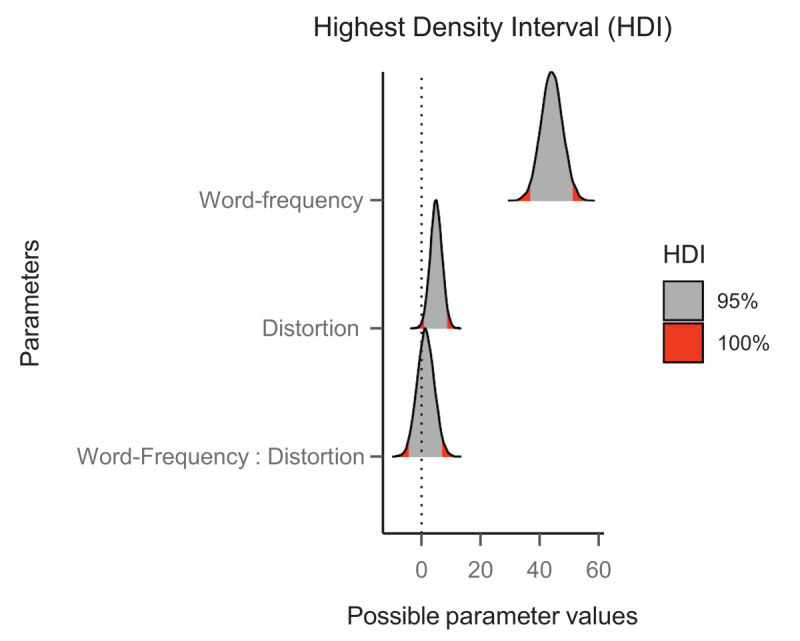
Posterior distributions in the latency data of Experiment 1. The grey areas represent the 95% credible intervals of each parameter estimate.

Each model was run for 5000 iterations (1000 for warmup), across four chains. In both models, the chains converged successfully, and all the values of R̂ (i.e., an index of convergence of the chains) were at their optimal value (i.e., 1.00). The output of the model provides the coefficient (b) of each estimate (this is the median of the posterior distribution), its standard error, and the 95% credible interval (95% CrI) of the estimate. We assumed evidence of an effect if the 95% CrI of its parameter estimate did not cross zero. The posterior distributions of each parameter, including the 95% credible intervals, on the latency data for words are shown in Figure 3. We employed the *emmeans* package (Lenth et al., 2021) for the simple tests’ effects in case of evidence of a word frequency by letter distortion interaction.

#### Word data

In the latency model, we observed faster response times for high-frequency than low-frequency words (*b* = 45.20, SE = 3.06, 95% CrI [39.20, 51.09]). We also found some evidence of a slight disadvantage for the shifted words (*b* = 5.63, SE = 1.48, 95% CrI [2.72, 8.54]) but no indication of an interaction between the factors (*b* = 1.44, SE = 2.89, 95% CrI [–4.22, 7.19]).

Regarding the accuracy model, we observed higher accuracy for high-frequency words than low-frequency words (*b* = –1.50, SE = 0.21, 95% CrI [–1.92, –1.13]). However, we did not find evidence of an effect of letter distortion (*b* = –0.21, SE = 0.19, 95% CrI [–0.58, 0.15]), nor did we find evidence for an interaction between word frequency and letter distortion (*b* = 0.62, SE = 0.33, 95% CrI [–0.01, 1.30]).

#### Nonword data

Participants responded faster when the nonwords were presented in their intact format than when the lower half was shifted (*b* = 4.00, SE = 1.73, 95% CrI [0.61, 7.35]). The accuracy data did not reveal any signs of an effect of letter distortion (*b* = –0.02, SE = 0.16, 95% CrI [–0.34, 0.27]).

The present experiment revealed a sizeable effect of word frequency (i.e., faster and more accurate responses to high- than low-frequency words). We also found a minimal, but consistent effect of letter distortion: the estimate of the effect was 4.95 ms. This difference was very similar to the reading cost when using harmonic means (5 ms)—note, however, that this cost was numerically lower when using the arithmetic means, probably due to their higher sensitivity to outliers (see Ratcliff, 1993). In addition, the pseudowords also showed a small cost due to letter distortion.

**Figure 4:**
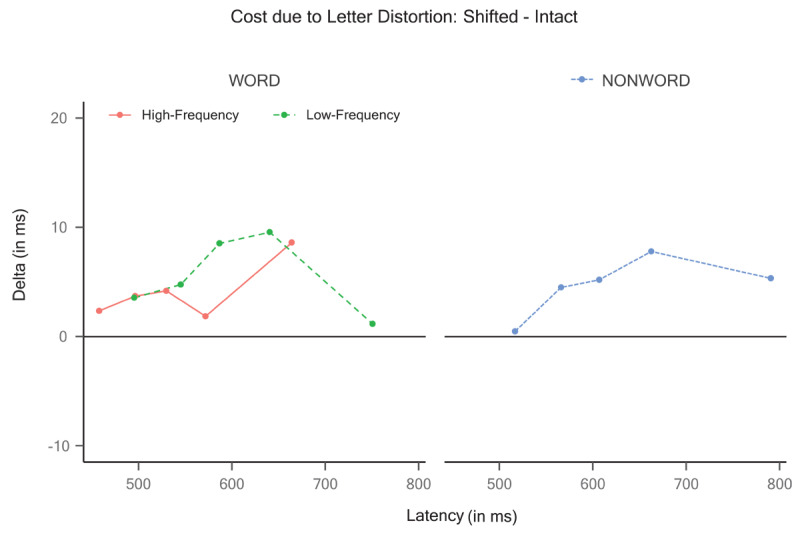
Exploratory quantile-based analyses using delta plots by computing the .1, .3, .5, .7, and .9 quantiles per subject in Experiment 1. The Y-axis reflects the reading cost (shifted – intact).

To supplement the effect of letter distortion on the word stimuli, we performed an exploratory quantile-based analysis using delta plots (e.g., see Schwarz & Miller, 2012) by computing the .1, .3, .5, .7, and .9 quantiles per subject and condition. Then, we calculated the difference between the shifted and intact conditions for the high-frequency and low-frequency words in each quantile. As shown in Figure 4, the delta plot revealed a minimal but consistent cost across quantiles of the shifted format relative to the intact format (e.g., all data points in the plots are above zero) for both words (left panel) and pseudowords (right panel).

Given the small size of the effect of letter distortion in the present experiment, the more sensible conclusion is that, despite the alteration in the vertical lines and curves of the word’s letter, the letter detectors in the word identification system are highly resilient to these modifications. It is noteworthy that a similar minimal cost has also been found in word identification times when comparing intact words and words in which their letters are rotated at low angles (22.5º, e.g., 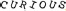, see Fernández-López et al., 2023).

The issue now is whether a larger manipulation of letter distortion (via a more significant displacement, e.g., 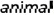) presents a challenge for the letter detectors—note that the junctions across the upper and lower halves in the distorted items are now more salient. We also examined whether the more pronounced level of letter distortion affects lexical processing (as indexed by the word-frequency effect). An interaction between the two factors would reveal some top-down feedback to normalize the percept. In this scenario, the reading cost due to letter distortion would be greater for low- than for high-frequency words—this pattern would resemble that reported when using difficult-to-read handwritten words (Barnhart & Goldinger, 2010; Vergara-Martínez et al., 2021) or words with rotated letters at angles of 67.5º (Fernández-López et al., 2023).

## Experiment 2: Moderate shift of the lower half

### Method

#### Participants

We recruited an additional sample of 78 participants (mean age = 26 years, SD = 4.7 years, 43 female) from the same population as Experiment 1.

#### Materials and Procedure

The materials and procedure were the same as in Experiment 1, except that the shift in the upper/lower parts of the items was larger (1.5 mm instead of 1 mm; e.g., 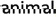; the corresponding example in Experiment 1 was 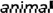).

### Results and Discussion

**Figure 5:**
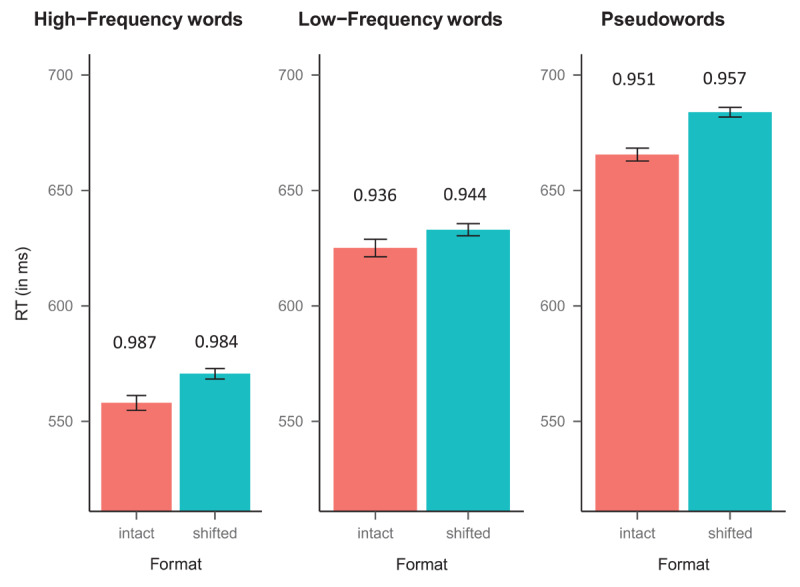
Mean response times (RTs, in ms) for the experimental conditions in Experiment 2. The error bars represent the standard errors, and the number above the bars correspond to the accuracy. *Note*: The values of the harmonic means were as follows: (1) high-frequency words (532 vs. 545 ms for the intact and shifted words, respectively); (2) low-frequency words (592 vs. 601 ms for the intact and shifted words, respectively); and (3) pseudowords (632 vs. 646 ms for the intact and shifted pseudowords, respectively).

**Figure 6:**
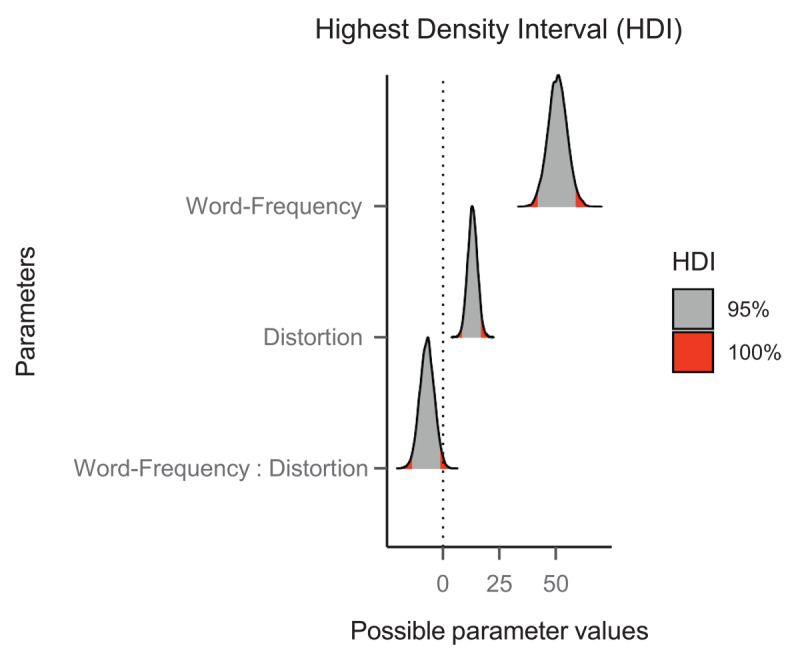
Posterior distributions in the latency data of Experiment 2. The grey areas represent the 95% credible intervals of each parameter estimate.

The analyses were the same as in Experiment 1. The percentage of excluded fast responses in the response time analyses was 0.01% for both word and nonword stimuli. Figure 5 presents the mean response times and accuracy per condition. The models provided an excellent fit to the data (R̂ = 1.00 in all cases). The posterior distributions for all the parameters are presented in Figure 6.

#### Word data

The model of response times showed faster responses to high- than low-frequency words (*b* = 46.28, SE = 3.45, 95% CrI [39.63, 53.11]). We also found evidence of a reading cost due to letter distortion (*b* = 9.55, SE = 1.72, 95% CrI [6.15, 12.87]) that was modulated by word frequency (interaction: *b* = –6.89, SE = 3.14, 95% CrI [–13.03, –0.80]). This interaction showed a greater effect of letter distortion for high-frequency words (*b* = –12.99, 95%CrI [–17.40, –8.73]) than low-frequency words (*b* = –6.14, 95%CrI [–10.90, –1.28]).

The model of accuracy revealed fewer error responses to high- than low-frequency words (*b* = –1.65, SE = 0.26, 95% CrI [–2.18, –1.16]). We did not find clear evidence of a reading cost due to letter distortion (*b* = –0.26, SE = 0.26, 95% CrI [–0.78, 0.23]) or of a two-way interaction (*b* = 0.38, SE = 0.28, 95% CrI [–0.15, 0.94]).

#### Nonword data

We found faster lexical decision times for the intact pseudowords than for the shifted pseudowords (*b* = 8.61, SE = 1.86, 95% CrI [4.97, 12.22]). The accuracy data showed 0.6% fewer errors for the shifted pseudowords than for the intact pseudowords (*b* = 0.31, SE = 0.14, 95% CrI [0.04, 0.60]).

Thus, the present experiment showed that a moderate shift in the upper/lower parts of a word’s letters hindered word processing relative to the intact format. Notably, the effect of letter distortion interacted with word frequency (13 vs. 7 ms for the high- and low-frequency words, respectively). In addition, we also found a reading cost of letter distortion for the pseudowords (18 ms).

**Figure 7:**
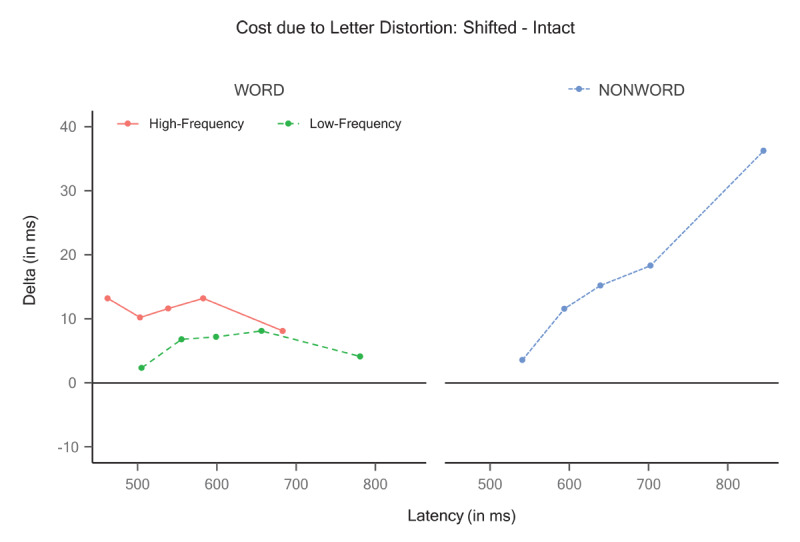
Exploratory quantile-based analyses using delta plots by computing the .1, .3, .5, .7, and .9 quantiles per subject in Experiment 2. The Y-axis reflects the reading cost (shifted – intact).

To complement these analyses, as in Experiment 1, we obtained the delta plots (see Figure 7), where the Y-axis again reflects the reading cost of the shifted relative to the intact items for the high- and low-frequency words (left panel. The delta plots showed some cost for the shifted format relative to the intact format, which was consistently larger across quantiles for high-frequency words than for low-frequency words (left panel). Furthermore, the pseudowords also showed a sizeable cost, which progressively increased across quantiles (right panel).

### General Discussion

Adult skilled readers can identify written words, regardless of their visual form (e.g., handwritten text, captchas), with relative ease (e.g., Grainger, 2022; Hannagan et al., 2012; Qiao et al., 2010). To explain this capability, researchers often propose that the letter detectors in our word recognition system have developed a tolerance to noise, a mechanism that would be shared with the detectors in neurobiological models of object recognition (e.g., Dehaene et al., 2005; Riesenhuber & Poggio, 1999). In the context of word identification and reading, allocating extra attentional resources for lexical access would become necessary only when the low quality of the visual input poses obstacles to automatic word processing (see Dehaene & Cohen, 2007; Qiao et al., 2010; Vergara-Martínez et al., 2021). However, as noted by Rey et al. (2009a), research on the intricacies of letter perception has often been disregarded in the front-end of computational models of visual word recognition (see also Perea et al., 2023, for review).

In the present experiments, we introduced a novel manipulation that shifted the lower half of letters in words. This modification altered the transition between the upper and lower halves of each letter, resulting in fragmented lines and curves via junctions that do not exist in the pristine forms. The shift was subtle in Experiment 1 (e.g., 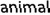) and moderate in Experiment 2 (e.g., 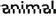). Experiment 1 revealed a small but reliable cost of letter distortion in the latency data, relative to the intact items, which affected similarly high- and low-frequency words. In Experiment 2, we found a larger cost of letter distortion, and this cost was shaped by word frequency (i.e., the cost of distortion was larger for high- than low-frequency words).

The neurobiological-inspired models proposed by Dehaene et al. (2005) and Grainger et al. (2008) provide a general framework that can account for this general pattern. When the degree of letter distortion is small (e.g., 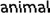, Experiment 1), the letter detectors in the word recognition system can respond nearly equally well as with the intact format, thus showing tolerance to distortion (the estimate was 4.95 ms). In Experiment 2, as the level of letter distortion increased to a moderate extent (e.g., 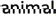), the visual integrity of the letters within the items was compromised to a greater extent. This distortion led to disruptions in the continuity of curves and lines and the creation of non-existent junctions. Notably, under these conditions, the effect of letter distortion exhibited a larger numerical magnitude, suggesting potential limitations to the resilience of letter detectors against distortion. Nonetheless, it is worth mentioning that even in Experiment 2, the reading cost remained consistently below 15 ms (see Figure 7) except for the .9 quantile in the pseudowords, which showed an increased cost.

Thus, our findings suggest that the letter detectors in the word recognition system exhibit a remarkable tolerance towards spurious junctions caused by shifted elements. Consequently, our results limit the critical role of viewpoint junctions in letter and word recognition (see Szwed et al., 2009). Had these junctions played a fundamental role for the letter detectors, the reading cost would have been significantly higher than the results observed in our experiments.

Notably, letter distortion appears to impact the level of whole-word representations in Experiment 2, as deduced from the interaction between letter distortion and word-frequency in the latency data. However, we exercise caution about drawing firm conclusions from this interaction: (1) the evidence supporting the interaction effect was numerically small (see Figures 5 and 6), and (2) the pattern itself was unexpected, revealing a greater impact of letter distortion on the response times to high-frequency words compared to low-frequency words.^3^ As mentioned in the Introduction, when comparing handwritten words with bad penmanship and printed words and when comparing intact words and words with letters rotated at high angles (e.g., 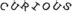), the reading cost in the latency data is consistently larger for low- than high-frequency words (e.g., Barnhart & Goldinger, 2010; Fernández-López et al., 2023; Perea et al., 2016; Vergara-Martínez et al., 2021). However, the reading cost due to letter distortion of Experiment 2 was greater for high- than low-frequency words. While admittedly *ad hoc*, an explanation for the interaction between letter distortion and word-frequency in Experiment 2 is that identifying high-frequency words may rely on holistic word processing (see Chen et al., 2016; Ventura et al., 2019, 2020). In this scenario, shifting the word’s lower letters could have hindered the holistic identification of highly frequent forms more than that of less frequent forms. We acknowledge that further research using a larger word-frequency range and more extreme manipulations of letter distortion would be necessary to examine the generality of this interaction. However, this research program would be beyond the scope of the current paper.

In conclusion, the present lexical decision experiments offer valuable insights into the remarkable resilience of letter detectors to distortion during by offsetting the lower part of the word’s letters (e.g., 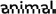). Notably, this manipulation offers researchers a parametric approach to manipulating the level of letter distortion (i.e., by varying the amount of shift, thereby altering the letters’ internal features) that differs from another manipulation of letter distortions such as letter rotation (which involves changing the orientation of letters without modifying their internal features). Furthermore, it can be combined with other techniques (e.g., masked priming; see Petit & Grainger, 2002) and dependent variables better suited for investigating online processing during letter/word recognition and reading, such as event-related potentials (e.g., see Grainger & Holcomb, 2009; Madec et al., 2012; Rey et al., 2009b) or eye fixation measures (see Blythe et al., 2019; Fernández-López et al., 2021). A final advantage of the current manipulation is that it may extend beyond letter and word identification, such as object and face recognition. For instance, it could be employed in face recognition tasks, enhancing our comprehension of the similarities and differences in perceptual and cognitive processes across different visual objects (e.g., words vs. faces; see Robotham & Starrfelt, 2017; Ventura, 2014).

## Data Accessibility Statement

The data files, scripts, and outputs are available at https://osf.io/s8cyk/?view_only=cbc0190333a74eb0833c66938c3d730d.

## Footnotes

We found out about the manipulation used by Wilson and Taylor (2009) when writing the draft of this paper. Unlike Wilson and Taylor (2009), our manipulation connected the upper and lower parts of the words’ letters.

At an informal level, offsetting some parts of the word’s letters is relatively common in advertisements or TV series (e.g., the series ), thus suggesting that readers can easily encode these words.

The accuracy data in Experiment 2 also showed trends towards a diverging pattern for high- and low-frequency words: While there was a small 0.3% cost for high-frequency words in the shifted format, low-frequency words showed a 0.8% benefit in the shifted format.
